# ﻿Multi-omics reveals nitrate-induced oxidative stress and morphogenesis pathways in *Morchella importuna*

**DOI:** 10.3897/imafungus.17.159999

**Published:** 2026-01-06

**Authors:** Yang Yu, Tianhai Liu, Jing Li, Xiang Wu, Shengyin Zhang, Yong Wang, Jie Tang, Weihong Peng, Francis M. Martin, Hao Tan

**Affiliations:** 1 National-Local Joint Engineering Laboratory of Breeding and Cultivation of Edible and Medicinal Fungi, Sichuan Institute of Edible Fungi, Sichuan Academy of Agricultural Sciences, Chengdu 610000, China Sichuan Institute of Edible Fungi, Sichuan Academy of Agricultural Sciences Chengdu China; 2 The National Key Laboratory of Ecological Security and Sustainable Development in Arid Region, Northwest Institute of Eco-Environment and Resources, Chinese Academy of Sciences, Lanzhou 730000, China Northwest Institute of Eco-Environment and Resources, Chinese Academy of Sciences Lanzhou China; 3 Université de Lorraine, INRAE, UMR Interactions Arbres/Microorganismes, Centre INRAE Grand Est - Nancy, Champenoux 54280, France Université de Lorraine Champenoux France

**Keywords:** Glutathione, homeostasis, NADP-glutamate, Nitrogen forms, oxidative stress, tyrosine

## Abstract

Nitrate (NO_3_^−^) and ammonium (NH_4_^+^) are the two main forms of inorganic nitrogen (N) found in soil. Most macrofungi show a preference for specific forms of N; however, the mechanisms behind these preferences remain poorly understood. In this study, we explored the metabolic responses induced by NO_3_^−^ and NH_4_^+^ uptake and assimilation in the ascomycete *Morchella importuna*, a highly valued soil-grown mushroom. Through transcriptomics, proteomics and metabolomics, we demonstrated that growth on NO_3_^−^ inhibited the expression and activity of NADP-glutamate dehydrogenase while increasing the expression and activity of glutamate synthase (GOGAT) and glutamate levels, underscoring the significant role of the GOGAT pathway in glutamate synthesis in NO_3_^−^-grown mycelia. Furthermore, growth on NO_3_^−^ results in the downregulation of proteins involved in ribosome biogenesis and RNA transport pathways, inducing a status analogous to N starvation and oxidative stress. Simultaneously, nitrate initiated metabolic alterations related to sexual morphogenesis, such as increased glutathione levels to counter oxidative stress, the upregulated expression of tyrosinase and its substrates to accelerate melanin deposition and enhanced glycosylation to supply cell-wall formation. These findings enhance our understanding of the differential response mechanisms to N sources that affect mushroom cell homeostasis.

## ﻿Introduction

Nitrogen (N) is crucial for fungal growth. Fungi can thrive in diverse environments and endure nutrient stress because of their ability to metabolise various nitrogen sources (Tudzynski 2014; Zhang et al. 2024b). In soil, nitrate (NO_3_^−^) and ammonium (NH_4_^+^) are the two primary inorganic N sources for fungi (Nehls and Plassard 2018). NH_4_^+^ is the preferred N source for most fungi, whereas NO_3_^−^ requires uptake via dedicated nitrate/nitrite transporters and energy-intensive reduction by nitrate reductase and nitrite reductase before entering the N assimilation pathway (Nygren et al. 2008; Clemmensen et al. 2013). In fungi, the assimilation of N from NH_4_^+^ directly or from NO_3_^−^ reduction mainly occurs through two primary pathways. The first is the glutamine synthetase (GS)/glutamate synthase (GOGAT) cycle: GS catalyses the incorporation of NH_4_^+^ into glutamate to form glutamine and GOGAT then transfers the amido group of glutamine to 2-oxoglutarate, yielding two glutamates. The second pathway involves NADP-dependent glutamate dehydrogenase (NADP-GDH), which catalyses the conversion of NH_4_^+^ and 2-oxoglutarate into glutamate (Martin et al. 1986; Martin et al. 1994). Different forms of nitrogen can significantly influence the growth and development of macrofungi. NH_4_^+^ is often supplemented in mushroom cultivation substrates as an active ingredient (Carrasco et al. 2018; Noble et al. 2024). Although most fungi tend to grow better on NH_4_^+^, they also exhibit a broad capability to utilise NO_3_^−^ (Nygren et al. 2008; Kohler et al. 2015). For instance, *Coprinopsis phlyctidospora* cultured in NO_3_^−^ exhibited higher mycelial biomass and more fruiting bodies than those cultured on NH_4_^+^ medium (He et al. 2003).

After more than a century of research, the morel mushroom (*Morchella* spp., *Morchellaceae*, *Pezizomycotina* and *Ascomycota*), which is renowned for its distinctive flavour and nutritional value, has recently been domesticated as a commercial crop (Du et al. 2020; Tao et al. 2025). The scale of *Morchella* cultivation is rapidly expanding globally; however, it remains highly unpredictable, as approximately 70% of farmers experience low yields or fruiting failure annually (Yu et al. 2021; Yu et al. 2025). Previous studies have attributed these outcomes to several factors, notably climatic conditions (Liu et al. 2023), deleterious soil microbes (Longley et al. 2019; Tan et al. 2021a; Yu et al. 2022b; Dong et al. 2023) and, more significantly, unsuitable soil physicochemical properties, with C, N, P and K levels being major contributing factors (Li et al. 2024; Zhang et al. 2024a). As a soil-cultivated edible fungus, the C source for cultivated *Morchella* is supplied by above-ground nutrition bags enriched with lignocellulose and other plant polysaccharides, which subsequently support the fructification of ascocarps in the soil bed (Tan et al. 2019). In contrast to C acquisition pathways, *Morchella* predominantly acquires N from the soil bed itself (Tan et al. 2019). Consequently, soil N fertilisers have a strong influence on *Morchella* fruiting. However, the mechanisms by which different forms of N regulate the physiological status of morel hyphae and thereby drive fruiting have not yet been elucidated.

Several studies have assessed the influence of soil nitrogen (N) on fruiting body formation in *Morchella*. Specifically, microorganisms involved in N fixation and nitrification have been correlated with increased morel yields (Yu et al. 2022a). Kang et al. (2026) employed machine-learning based correlation analysis and identified soil NO_3_^−^ concentration as a key booster of morel yield. A comprehensive investigation demonstrated that morel yields exhibit a significant positive correlation with soil NO_3_^−^ content and a significant negative correlation with soil NH_4_^+^ content (Zhang et al. 2024a). When utilised as the primary N source in the fruiting substratum, NO_3_^−^ has been found to be more favourable for the formation of morel-fruiting bodies than NH_4_^+^ (Tan et al. 2021b; Li et al. 2024). This observation suggests that NO_3_^−^ is more advantageous for the formation of morel-fruiting bodies than NH_4_^+^. However, the regulatory mechanisms involved in N acquisition and assimilation during morphogenesis in *Morchella* remain unclear.

We hypothesised that feeding *Morchella* with different N forms would activate distinct N assimilation pathways and downstream metabolic responses during its vegetative growth, leading to divergent physiological states. To investigate the distinct metabolic responses occurring in the morel mycelium in response to NO_3_^−^ and NH_4_^+^, we used *Morchella importuna* grown on either NO_3_^−^ or NH_4_^+^ as the sole N source. Integrated transcriptomic, proteomic and metabolomic analyses were conducted to examine alterations in gene expression and metabolite levels in *M. importuna* mycelia to elucidate the differential responses of inorganic N utilisation. Our data demonstrated that NO_3_^−^ and NH_4_^+^ induced metabolic alterations related to sexual morphogenesis and established distinct cellular homeostasis in *Morchella* by activating different N assimilation pathways. These findings contribute to our understanding of how different N forms influence the growth and development of *Morchella* and provide a scientific foundation for promoting morel productivity through the modulation of N nutrition.

## ﻿Materials and methods

### ﻿Experimental design

**Figure 1.**
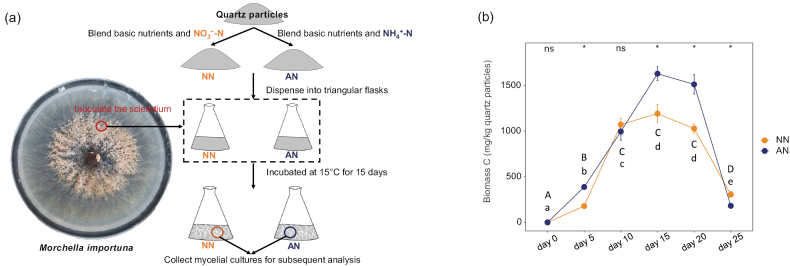
**a** experimental design diagrams for cultivating *Morchella importuna* under different N forms **b** growth curves of *Morchella importuna* in NO_3_^−^-N (NN) and NH_4_^+^-N (AN) treatments. Capital and lowercase letters respectively represent the differences between different time points in NN and AN. The same letters indicate no significance (P > 0.05). * indicates a significant difference (P < 0.05) between NN and AN at the same time point.

To mimic soil conditions, two culture media were formulated with quartz particles as the inorganic matrix (Tan et al. 2021b); all nutrients blended into the matrix were identical, except for the N source. One medium contained NO_3_^−^-N (NN), while the other contained NH_4_^+^-N (AN) as the sole N source. The N concentrations were set to approximate previously measured soil N levels in large-scale morel cultivation sites across China (Tan et al. 2021a; Chen et al. 2025). The prepared NN and AN media were dispensed into triangular flasks. Then, the sclerotium of *M. importuna* was inoculated into the NN and AN media. According to the typical soil temperature during the field cultivation, the inoculated *M. importuna* was then incubated at 15 °C. Quartz particle samples with mycelia were then collected, snap-frozen in liquid nitrogen and stored at −80 °C (Thermo Fisher Scientific, Waltham, MA, USA) for subsequent analysis (Fig. 1a).

### ﻿Medium design

The *M. importuna* culture medium was prepared according to previous methods with some modifications (Tan et al. 2021b). The modified medium was formulated by blending 20 g of glucose, 20 g of starch, 4 g of anhydrous calcium sulphate, 1 g of potassium dihydrogen phosphate, 0.1 g of magnesium sulphate and 0.01 g of sodium chloride into 1000 g of quartz particles with a diameter range between 0.18 and 0.25 mm. The medium was thoroughly mixed and then divided into two equal portions. The commonly adopted water-soluble inorganic N sources, namely KNO_3_ and (NH_4_)_2_SO_4_, were added to the NN and AN treatment, respectively. The final concentration of nitrogen in each group was 2 × 10^−5^ mol/g. A trace element supplement solution was prepared by dissolving 4 g of ferric chloride, 1 g of boric acid, 0.15 g of manganese sulphate, 0.15 g of zinc sulfate and 0.04 g of copper sulphate in 500 ml of distilled water. To each 500 g of NN and AN medium, 250 µl of the trace element supplement solution was added, followed by 400 ml of distilled water to adjust the moisture content of the medium. The NN and AN media were then evenly dispensed into five 500 ml triangular flasks, which were subsequently sterilised at 115 °C for 30 min and stored for further use.

### ﻿Strain culture and phenotypic observation

In this study, a dikaryotic *M. importuna* (SCYDJ1-A1), deposited in the Germplasm Resource Center Bank of Sichuan Province, China, was used as the culture strain. This strain has been widely cultivated on a commercial scale in China (Tan et al. 2019). Then, a single sclerotium of *M. importuna* was inoculated into NN and AN media, with five replicates for each medium. The inoculated *M. importuna* was then incubated at 15 °C. Samples of quartz particles were collected on days 5, 10, 15, 20 and 25 days post-inoculation (DPI). The microbial biomass C of the quartz particles were measured by CHCl_3_-fumigation method, as described by Vance et al. (1987). The mycelial phenotype of *M. importuna* in quartz particles was observed using a DMV6 stereomicroscope (Leica, Wetzlar, Germany). Based on the phenotypic observations, quartz particle samples with mycelia collected from 15 DPI were selected for subsequent multi-omics and biochemical experiments.

### ﻿RNA extraction

Total RNA from the NN and AN samples was isolated and purified using TRIzol reagent (Invitrogen, Carlsbad, CA, USA), according to the manufacturer’s instructions. A NanoDrop spectrophotometer (Thermo Fisher Scientific, Waltham, MA, USA), Qubit RNA Kit (Life Technologies, Carlsbad, CA, USA), and 2100 Bioanalyzer (Agilent Technologies, Santa Clara, CA, USA) were used to determine the purity, concentration and integrity of each sample, respectively.

### ﻿cDNA library construction, sequencing and differential expression analysis

The mRNA was enriched using oligo (dT)-conjugated magnetic beads. The mRNA was randomly fragmented by adding fragmentation buffer. First-strand cDNA was synthesised using random hexamers as primers with mRNA as the template. Second-strand cDNA was synthesised by adding the buffer, dNTPs and DNA polymerase. AMPure XP beads were used to purify the double-stranded cDNA. The ends of the purified double-stranded cDNA were repaired and a tail and sequencing adapter were added. AMPure XP beads were used to select the fragment size and the final cDNA library was enriched using PCR. After construction, the cDNA library was assessed for insert size and effective concentration to ensure the library quality. Sequencing was performed on an Illumina HiSeq 4000 platform (Illumina, San Diego, CA, USA). The FASTQ QualityFilter tool from the FASTX toolkit was used to filter raw data. HISAT2 software was used to map the reads to the completed *M. importuna* genome sequence (available at the DOE-JGI website: https://genome.jgi.doe.gov/portal/Morimp1/Morimp1.download.html) during RNA-seq data pre-processing (Tan et al. 2019). The fragments per kilobase million (FPKM) were used to normalise transcript abundance. Differential expression analysis between the NN and AN treatments was performed using the DESeq2 R package (version 1.16.1). The resulting *P*-values were adjusted using the Benjamini and Hochberg’s approach to control the false discovery rate (Benjamini and Hochberg 2018). Genes were considered to be differentially expressed genes (DEGs) when the |fold change| > 2, with an adjusted *P*-value < 0.05, between the NN and AN treatments, as described in a previous study (Chen et al. 2025).

### ﻿Protein extraction and digestion

Proteins were extracted from all the samples using SDS-DTT-Tris buffer (4% SDS, 100 mM Tris-HCl and 1 mM DTT, pH 7.6). Protein concentrations were quantified using a BCA Protein Assay Kit (Bio-Rad, Hercules, CA, USA). Proteins were then digested using the filter-aided sample preparation (FASP) protocol, described by Wiśniewski et al. (2009). The digested peptides from each sample were desalted on an Empore™ C18 column (standard density, bed diameter 7 mm, volume 3 ml, Sigma, Saint-Quentin Fallavier, France), concentrated by vacuum centrifugation and reconstituted in 40 μl formic acid (0.1%).

### ﻿Tandem mass tag (TMT) labelling and high-performance liquid chromatography–tandem mass spectrometry (HPLC–MS/MS) for proteomics

The peptides were then processed using the TMT kit (Thermo Fisher Scientific, Waltham, MA, USA) according to the manufacturer’s protocol (Kamińska et al. 2025). High-pH reverse-phase HPLC was performed using an Agilent 300 Extend C18 column (250 mm × 4.6 mm, 5 μm, Agilent, Santa Clara, CA, USA) to fractionate the tryptic peptides into 18 fractions. The peptides were separated into 80 fractions using an acetonitrile gradient (2–60%) in 10 mM ammonium bicarbonate at pH 10 over 80 min. These fractions were then combined into 10 pools and dried using vacuum centrifugation. Each condition was replicated thrice. A total of 10 TMT runs were performed using nine TMT tags per run (126, 127 N, 128 N, 129 N, 130 N, 131N, 132 N, 133 N, 134 N, 135 N).

LC-MS/MS analysis was performed using a timsTOF Pro mass spectrometer (Bruker, Houston, TX, USA) coupled with a NanoElute system (Bruker, Houston, TX, USA) and 60/120/240 min. The peptides were loaded on to an Acclaim PepMap100 reverse-phase trap column (100 μm × 2 cm, Thermo Fisher Scientific, Waltham, MA, USA) connected to a C18-reversed-phase analytical column (10 cm × 75 μm, 3 μm resin, Thermo Fisher Scientific, Waltham, MA, USA). The peptides were then separated in buffer A (0.1% formic acid) using a linear gradient of buffer B (84% acetonitrile and 0.1% formic acid) at a flow rate of 300 nl/min, controlled by the IntelliFlow technology. The mass spectrometer was operated in positive ion mode with a mass range of *m/z* 100–1700 and an ion mobility range of 1/K0 = 0.6–1.6. Parallel accumulation–serial fragmentation MS/MS were performed with a target intensity of 1.5 k and a threshold of 2500. Active exclusion was enabled with a release time of 0.4 min.

### ﻿Identification of the protein species

MaxQuant software (version 1.5.3.17) was used to combine and search raw MS data for each sample, and both identification and quantitative analyses were performed. The following parameters were applied: enzyme, trypsin; maximum missed cleavages, 2; fixed modifications, carbamidomethyl (C); variable modifications, oxidation (M); main search tolerance, 6 ppm; first search tolerance, 20 ppm; and MS/MS tolerance, 20 ppm. The false discovery rate (FDR) for protein and peptide identification was set to ≤ 0.01. Razor and unique peptides were used for peptide assignment and protein quantification, respectively. The match time window between runs was set to 2 min. Proteins were considered differentially expressed proteins (DEPs) when the |fold change| > 1.5 between NN and AN treatments with a *P*-value < 0.05, as described by Fu et al. (2023).

### ﻿Metabolite extraction

Each sample (1 g) of quartz particles with mycelia was slowly thawed at 4 °C, followed by the addition of 5 ml of 80% methanol–water solution and the mixture was vortexed for 30 s to form the metabolite extraction solution. The solution was then sonicated at 4 °C for 10 min. After centrifugation (12,000 × *g*, 10 min, 4 °C), the supernatant was collected and vacuum-dried. The residue was re-suspended in 1 ml of 10% methanol–water solution for LC-MS/MS analysis. Equal aliquots of the solution obtained from individual samples were combined to create quality control (QC) samples. QC samples were analysed after every six test samples (n = 6). Stability was assessed, based on the consistency of the QC samples during sample testing.

### ﻿LC-MS/MS analysis for metabolomics

Ultra-performance liquid chromatography (UPLC)–Orbitrap–MS system (Thermo Fisher, Waltham, MA, USA) using a Waters HSS T3 column (100 mm × 2.1 mm, 1.8 μm) was employed for LC-MS/MS analysis. The analytical conditions were set as follows: column temperature, 40 °C; flow rate, 0.3 ml/min and injection volume, 2 μl. The solvent system consisted of water (0.1% formic acid) and acetonitrile (0.1% formic acid) and the gradient programme was as follows: 0 min, 0% B; 2 min, 0% B; 14 min, 95% B; 14 min, 95% B; 13.8 min, 0% B; and 18 min, 0% B. High-resolution MS data were recorded on a Q Exactive HFX hybrid quadrupole-Orbitrap mass spectrometer, equipped with an ESI heating source (Thermo Fisher Scientific, Waltham, MA, USA) using the Full-ms-ddMS2 MS acquisition method with a resolution of 70,000 full width at half maximum (FWHM). A positive/negative-ion scanning mode was used. The resolution of the data-dependent scan was 18,000 FWHM (Xu et al. 2021). The ESI source parameters were as follows: the spray voltage was −2.6 kV/2.8 kV; the sheath gas pressure was 38 arb; the aux gas pressure was 10 arb; the sweep gas pressure was 0 arb; the capillary temperature was 315 °C; and the aux gas heater temperature was 350 °C (Dunn et al. 2011; Doppler et al. 2016).

### ﻿Data analysis and interpretation for metabolomics

The raw MS data were acquired using Xcalibur software (version 4.1) on a Quadrupole-Exactive instrument (Thermo Fisher Scientific, Waltham, MA, USA). Progenesis QI software (Waters Corporation, Milford, MA, USA) was used for data pre-processing (Cai et al. 2015). A local database and commercial databases (https://www.hmdb.ca/ and https://metlin.scripps.edu/) were used to match the metabolite information. The detection system was considered robust and stable, with the generated data deemed reliable when more than 70% of the potential peaks in the QC samples had a relative standard deviation (RSD) of less than 30%. After normalisation, orthogonal partial least-squares discriminant analysis (OPLS-DA) was used to analyse the processed data. The robustness of the model was assessed using a seven-fold cross-validation and permutation testing. The contribution of each variable to the classification was evaluated by calculating the variable importance in projection (VIP) value in the OPLS-DA model. Student’s *t*-test was used to calculate the significance of the differences between the two treatments. Metabolites with VIP > 1, |fold change| > 1.5 and *P*-values < 0.05 were considered significantly differentially accumulated metabolites (DAMs), as described by Liu et al. (2024).

### ﻿Bioinformatics analysis

The annotation of the DEGs, DEPs and DAMs was conducted using Blast2GO software (https://www.blast2go.com/) and KAAS software (http://www.genome.jp/tools/kaas/). Gene set enrichment analysis (GSEA) (www.broad.mit.edu/gsea/msigdb/) was employed to explore the specific expression of mRNA and translated proteins of genes, as well as the metabolomic sets between NN and AN treatments.

### ﻿Biochemical assays

The enzymatic activities of all samples, including cellulase, xylanase, laccase, manganese peroxidase, lignin peroxidase, glucoamylase, lipase, protease and glucose dehydrogenase, were characterised according to the biochemical assay procedures described by Chen et al. (2025). Additionally, malondialdehyde (MDA), hydrogen peroxide (H_2_O_2_) and superoxide anion (O_2_^−^) were measured using the MDA-2-Y, H_2_O_2_-2-Y and SA-2-G detection kits (Keming Biotechnology Co., Ltd., Suzhou, China), respectively, following the manufacturer’s instructions.

### ﻿Statistical analysis

Student’s *t*-test was used to compare the ammonium- and nitrate-supplemented treatments, whereas analysis of variance (ANOVA) was conducted used to compare multiple time points in the ammonium- or nitrate-supplemented treatment. Differences were con­sidered statistically significant at a threshold of *P* < 0.05. Genes, proteins and metabolites between NN and AN treatments were considered differentially expressed or accumulated when the absolute fold change was (|FC|) > 2, 1.5 and 1.5, respectively and when *P* < 0.05. Significantly enriched KEGG pathways were identified using the limma package, with a significance threshold of *P* < 0.05.

## ﻿Results

### ﻿Mycelial growth of *M. importuna* on NO_3_^−^ or NH_4_^+^

To dynamically monitor the growth rate of *M. importuna* in NN and AN media, the CHCl_3_-fumigation method was used to detect the biomass C of *M. importuna* at 5, 10, 15, 20 and 25 days post inoculation (DPI) (Fig. 1b). The results indicated that the biomass C of NN treatment peaked at 10 DPI, while that of NN treatment reached its peak at 15 DPI. The biomass of both treatments began to decline at 20 DPI. The peak biomass of NN media was significantly lower than that of AN media (P < 0.05). Stereomicroscopic observation revealed that, when the biomass of both media was at its peak (15 DPI), their mycelia showed no significant phenotypic differences (Suppl. material 1).

### ﻿Differentially expressed transcripts between NO_3_^−^ and NH_4_^+^

**Figure 2.**
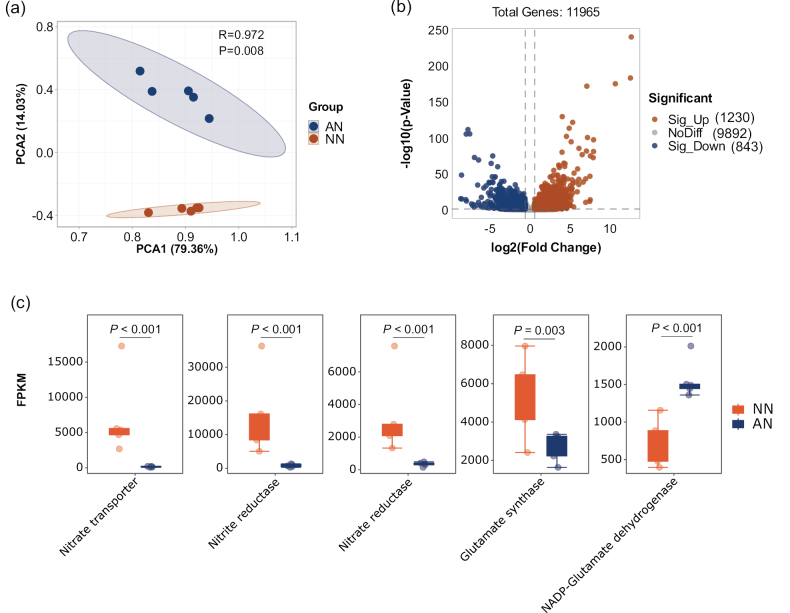
**a** PCA showing inter-group variations and intra-group consistency amongst transcriptome samples for NO_3_^−^-N (NN) and NH_4_^+^-N (AN) treatments **b** volcano plot depicting differentially expressed genes (DEGs) between the NO_3_^−^-N (NN) and NH_4_^+^-N (AN) treatments **c** transcriptional expression of key N-assimilation genes.

Transcript profiles of *M. importuna* mycelia grown on either NO_3_^−^ or NH_4_^+^ medium as the sole N source were obtained. Additionally, nearly 48 million reads were generated for 10 samples, with an average sequence alignment efficiency of ~ 85.5% (Suppl. material 3: table S1). PCA confirmed significant inter-group variance and intra-group reproducibility between NO_3_^−^ and NH_4_^+^ treatments (R = 0.972, *P* = 0.008) (Fig. 2a). In total, 11,965 genes were expressed, of which 2,073 (17.3%) were identified as DEGs between the NO_3_^−^ and NH_4_^+^ treatments (Suppl. material 3: table S2). On NO_3_^−^, 843 genes were upregulated, whereas 1,230 DEGs were downregulated (Fig. 2b).

Several genes involved in NO_3_^−^ uptake and assimilation were identified in NO_3_^−^ or NH_4_^+^ medium, including those encoding nitrate transporter (NRT), nitrate reductase (NR), nitrite reductase (NiR), glutamine synthetase (GS), glutamate synthase (GOGAT), NAD- and NADP-glutamate dehydrogenases (GDH), asparagine synthetase (ASN) and alanine aminotransferase (Suppl. material 3: table S3). As expected, *NRT*, *NR* and *NiR*, involved in NO_3_^−^ uptake and reduction, were upregulated in NO_3_^−^ medium (all fold changes > 8, all *P* < 0.001), whereas *NADP-GDH* was downregulated in NO_3_^−^ medium (fold change > 2, *P* < 0.001) (Fig. 2c). In addition, *GOGAT* was marginally upregulated in NO_3_^−^, suggesting a potential N assimilation role in NO_3_^−^ (fold change = 1.9, *P* = 0.003). This confirmed that the addition of different forms of N initiated distinct regulatory pathways for N assimilation in the *Morchella* hyphae.

### ﻿Functional gene modules enriched by NO_3_^−^

**Figure 3.**
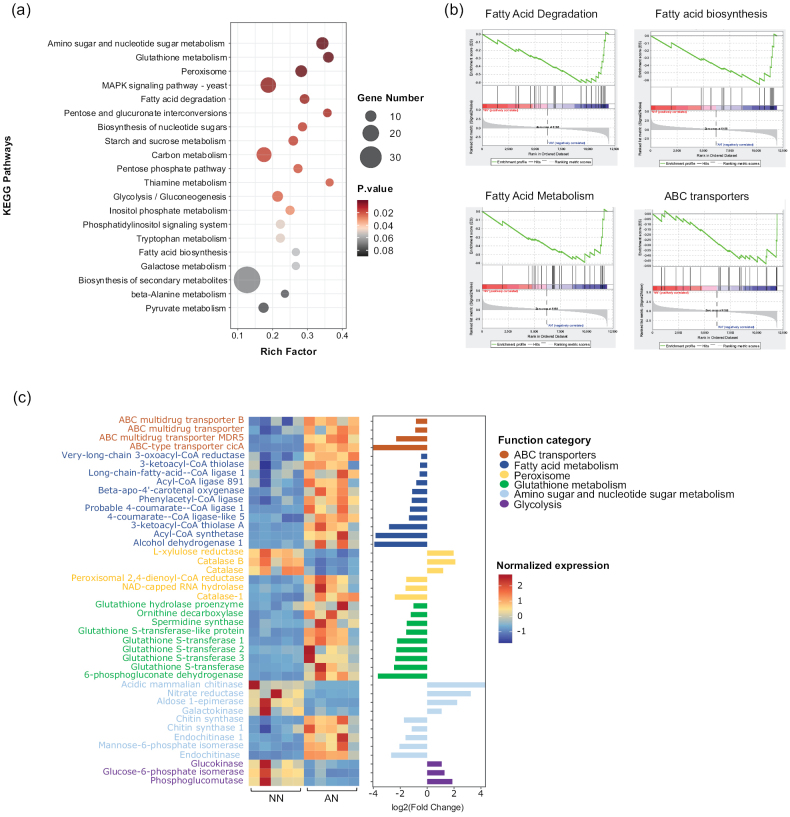
**a** KEGG enrichment analysis of DEGs between NO_3_^−^-N (NN) and NH_4_^+^-N (AN) treatments. The x-axis represents the enrichment factor, the circle size indicates the number of DEGs involved in the pathway and the colour gradient on the right represents the *P*-values of the KEGG pathway **b**GSEA revealing significantly enriched gene sets **c** heat map and bar chart illustrating functional genes identified by KEGG enrichment analysis and GSEA. The colour gradient on the right represents the normalised transcript expression levels.

KEGG enrichment analysis of the 2,073 DEGs between the NO_3_^−^ and NH_4_^+^ media identified three significantly enriched pathways (P < 0.05): amino sugar and nucleotide sugar metabolism, glutathione metabolism and peroxisome, with 12, 9 and 11 DEGs associated with these pathways, respectively (Fig. 3a, Suppl. material 3: table S4). GSEA was conducted on all 11,965 genes of NO_3_^−^ or NH_4_^+^ media with annotations from the KEGG database. Four gene sets were significantly downregulated (P < 0.05) in NO_3_^−^ treatment (Fig. 3b, Suppl. material 3: table S5), namely those related to fatty acid degradation, biosynthesis and metabolism and ABC transporters, with ten, six, nine and four core genes identified within these metabolic pathways, respectively. This finding suggests that changes in the N source induce dramatic rewiring of the major metabolic pathways, encompassing N, C and fatty acid metabolism, as well as oxidative-stress responses in *M. importuna*.

By integrating all the DEGs of enriched pathways and all core genes of enriched gene sets, a total of 42 genes were screened and defined as key genes (Fig. 3c). This includes four ABC transporters, 11 genes involved in fatty acid metabolism, six in peroxisome, nine in glutathione metabolism, nine in amino sugar and nucleotide sugar metabolism and three in glycolysis. Glucokinase, phosphoglucomutase and glucose-6-phosphate isomerase, which are related to glycolysis, are upregulated by NO_3_^−^. Similarly, two catalases and one L-xylulose reductase gene from the peroxisome were also induced by NO_3_^−^. In contrast, two endochitinases and two chitin synthases were downregulated by NO_3_^−^. Key genes involved in ABC transporters, glutathione metabolism and fatty acid metabolism were downregulated by NO_3_^−^.

### ﻿Differentially expressed proteins (DEPs) between NO_3_^−^ and NH_4_^+^

**Figure 4.**
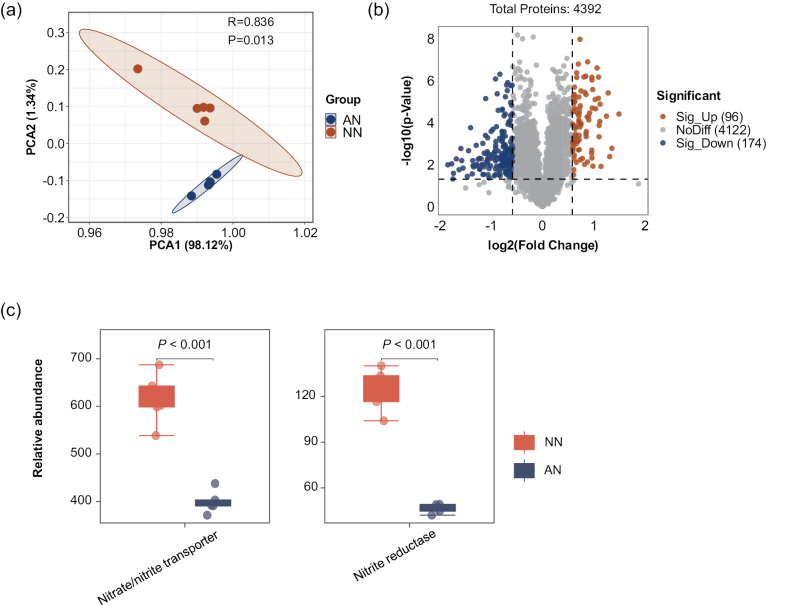
**a** PCA showing inter-group variations and intra-group consistency amongst proteomic samples from mycelia grown in NO_3_^−^-N (NN) or NH_4_^+^-N (AN) media **b** volcano plot illustrating the DEPs between NO_3_^−^-N (NN) and NH_4_^+^-N (AN) treatments **c** expression patterns of N assimilation proteins.

TMT-based quantitative proteomic profiling was used to assess the differential expression of proteins (DEPs) in *M. importuna* cultivated with either NO_3_^−^ or NH_4_^+^ as the sole N source. PCA confirmed significant intergroup differences and intragroup reproducibility between NO_3_^−^ and NH_4_^+^ treatments (R = 0.836, P = 0.013) (Fig. 4a). A total of 4,392 proteins were identified, including 270 DEPs (6%) (Suppl. material 3: table S6). Amongst these, 174 DEPs were upregulated in the NO_3_^−^ treatment, while 96 DEPs were downregulated compared with the NH_4_^+^ treatment (Fig. 4b). Amongst all identified proteins, 10 were involved in N uptake and assimilation, including NRT, two NiR, three GS, one GOGAT, two GDH and one ASN (Suppl. material 3: table S7). Consistent with the results of transcriptome analysis, two proteins involved in NO_3_^−^ reduction, NRT (fold change = 1.5, *P* < 0.001) and NiR (fold change = 2.7, *P* < 0.001), were upregulated after NO_3_^−^ treatment (Fig. 4c).

### ﻿Functional protein modules enriched by NO_3_^−^

**Figure 5.**
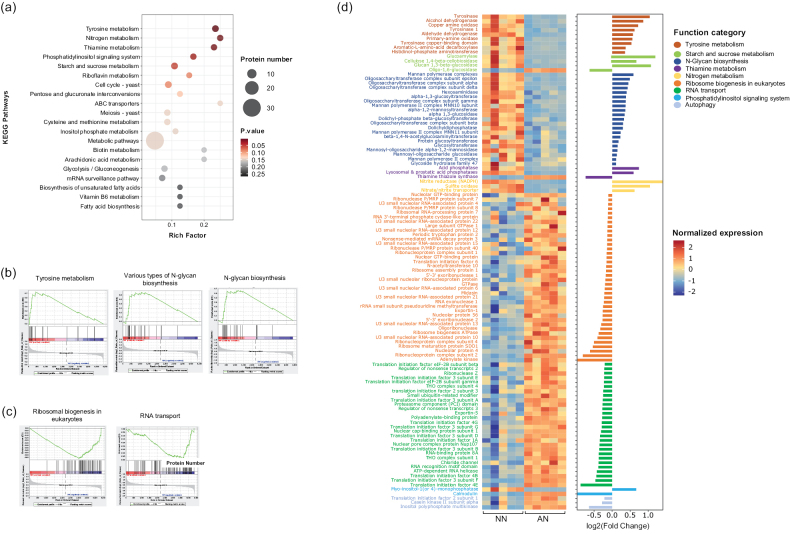
**a** KEGG enrichment analysis of DEPs from mycelia grown in NO_3_^−^-N (NN) or NH_4_^+^-N (AN) media. The x-axis represents the enrichment factor, the dot size indicates the number of DEPs involved in the pathway and the colour bars on the right represent the *P*-values of the KEGG pathway. GSEA revealed significantly enriched protein sets following **b** NO_3_^−^-N (NN) and **c** NH_4_^+^-N (AN) treatment **d** heat map and bar chart illustrating the functional proteins identified by KEGG enrichment analysis and GSEA. The colour bars on the right represent normalised protein expression levels.

KEGG enrichment analysis of the DEPs between the NO_3_^−^ and NH_4_^+^ treatments identified five regulated pathways (P < 0.05) (Fig. 5a): N, tyrosine, thiamine, starch, sucrose metabolism and the phosphatidylinositol signalling pathway. GSEA of the 4,392 identified proteins in the NO_3_^−^ or NH_4_^+^ media resulted in three protein sets that were upregulated by NO_3_^−^ treatment (P < 0.05) (Fig. 5b, Suppl. material 3: table S8), specifically tyrosine metabolism, various types of N-glycan biosynthesis and N-glycan biosynthesis, with 9, 16 and 15 core proteins identified within these sets, respectively. Two protein sets identified by GSEA were downregulated by NO_3_^−^ treatment (P < 0.05) (Fig. 5c, Suppl. material 3: table S9), ribosome biogenesis and RNA transport, with 39 and 30 core proteins identified within these sets, respectively. These results suggest that the form of N also influences the pathways involved in regulating gene and protein expression as well as the determination of cell fate.

A total of 111 proteins were screened by integrating all the DEPs from enriched pathways and all core genes in enriched protein sets, which were defined as key proteins (Fig. 5d). Amongst these, three, nine and 21 upregulated proteins in the NO_3_^−^ were involved in N metabolism, tyrosine metabolism and N-glycan biosynthesis, respectively. These included six oligosaccharyl transferases, four mannan polymerases and three tyrosinases. Furthermore, 37 and 28 proteins associated with ribosome biogenesis and RNA transport, respectively, were down-regulated by NO_3_^−^ treatment. These comprised 15 translation initiation factors, five ribonucleases, three nuclear GTP-binding proteins, three H/ACA ribonucleoproteins and nine U3 small nucleolar RNA-associated proteins, suggesting a suppression of the core translational machinery in response to NO_3_^−^.

### ﻿Differential accumulation of metabolites between NO_3_^−^ and NH_4_^+^

**Figure 6.**
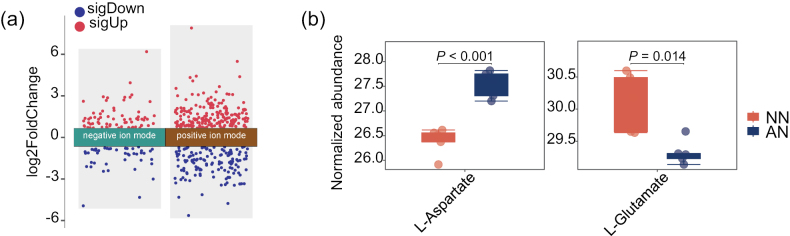
**a** volcano plot illustrating the differentially accumulated metabolites (DAMs) between NO_3_^−^-N (NN) and NH_4_^+^-N (AN) treatments **b** abundance of metabolites related to N assimilation pathways.

This study further investigated the effects of NO_3_^−^ and NH_4_^+^ on metabolite concentrations in *M. importuna* (Fig. 6a). A total of 296 metabolites were identified in the LC/MS negative ion mode, with 72 metabolites exhibiting a higher concentration and 45 demonstrating a lower level following NO_3_^−^ treatment than following NH_4_^+^ treatment (Suppl. material 3: table S10). In the positive ion mode, 824 metabolites were identified, with 202 upregulated and 45 downregulated metabolites in NO_3_^−^ treatment group (Suppl. material 3: table S11). Screening of the metabolites of the N assimilation pathway by combining the negative and positive ion modes revealed that L-glutamate was enriched (VIP = 1.2, fold change = 1.8, *P* = 0.01), whereas L-aspartate was found at lower concentrations (VIP = 1.4, fold change = 2.2, *P* < 0.001) in response to NO_3_^−^ (Fig. 6b, Suppl. material 3: table S12). The increased concentration of glutamate also confirmed the upregulation of *GOGAT* in transcriptomic analysis.

### ﻿Functional metabolism modules enriched by NO_3_^−^

**Figure 7.**
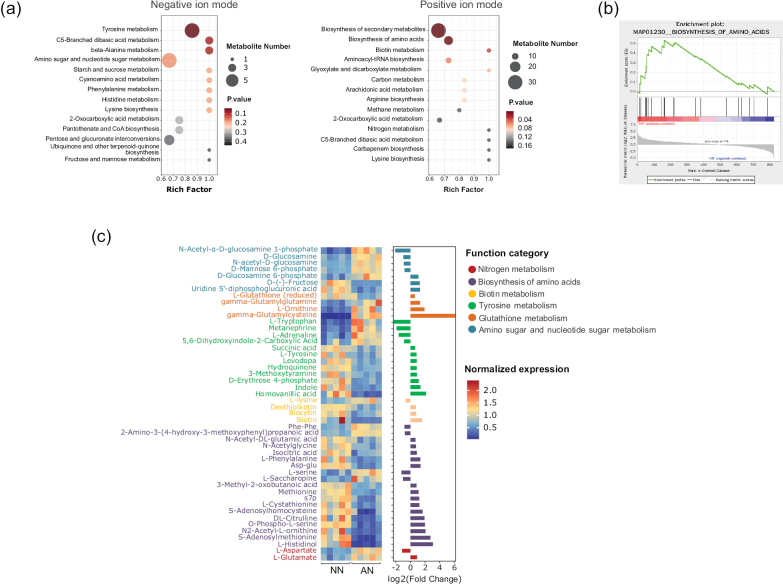
**a** KEGG enrichment analysis of differentially accumulated metabolites (DAMs) in mycelia grown on NO_3_^−^-N (NN) or NH_4_^+^-N (AN). The x-axis represents the enrichment factor, the dot size indicates the number of DAMs involved in the pathway and the colour bars on the right represent the *P*-values of the KEGG pathway **b**GSEA revealing significantly enriched metabolite sets **c** heat map and bar chart depicting functional metabolites identified using KEGG enrichment analysis and GSEA. The colour bars on the right represent normalised metabolite levels.

KEGG enrichment analysis of DAMs between the NO_3_^−^ and NH_4_^+^ treatments showed that the pathways of tyrosine metabolism, biosynthesis of secondary metabolites, amino acids and biotin metabolism were enriched (P < 0.05) (Fig. 7a, Suppl. material 3: table S13). GSEA of metabolites in the NO_3_^−^ or NH_4_^+^ revealed that the metabolite sets for amino acid biosynthesis and metabolic pathways were significantly upregulated by the NO_3_^−^ treatment (P < 0.05) (Fig. 7b, Suppl. material 3: table S14), with 12 and 53 core metabolites identified in amino acid biosynthesis and metabolic metabolite sets, respectively.

Our study screened metabolites in pathways, such as tyrosine metabolism, biosynthesis of amino acids and biotin metabolism, which were identified, based on metabolomic enrichment analysis, as well as metabolites in the amino sugar and nucleotide sugar metabolism, glutathione metabolism, nitrogen metabolism, starch and sucrose metabolism and thiamine metabolism pathways, which were identified through transcriptomic or proteomic analyses. These were identified as key metabolites, yielding 48 key metabolites. Amongst these, four metabolites involved in glutathione metabolism were upregulated under NO_3_^−^ treatment, including reduced glutathione (VIP = 1.1, fold change = 1.5, *P* = 0.03) and its precursor gamma-glutamylcysteine (VIP = 1.5, fold change = 73.0, *P* < 0.001). Notably, gamma-glutamylcysteine was the most upregulated metabolite after NO_3_^−^ treatment, demonstrating the key role of glutathione in response to NO_3_^−^. Nineteen metabolites involved in the biosynthesis of the amino acid pathway were identified, of which 15 were upregulated under NO_3_^−^ treatment. In addition, several metabolites involved in melanin synthesis, including L-tyrosine (VIP = 1.1, fold change = 1.8, *P* = 0.04) and L-dopa (VIP = 1.3, fold change = 1.8, *P* = 0.007), were upregulated by NO_3_^−^. Metabolites associated with amino sugar metabolism, such as fructose (VIP = 1.5, fold change = 2.4, *P* < 0.001), D-glucosamine 6-phosphate (GlcN-6-P) (VIP = 1.2, fold change = 2.1, *P* = 0.01) and uridine 5’-diphosphoglucuronic acid (UDP-GlcA) (VIP = 1.3, fold change = 2.5, *P* = 0.002), were upregulated by NO_3_^−^, whereas N-acetyl-α-D-glucosamine 1-phosphate, D-glucosamine, N-acetyl-D-glucosamine and D-mannose 6-phosphate were downregulated by NO_3_^−^. These metabolites act as regulatory factors for the synthesis of chitin or other fungal cell wall polysaccharides, demonstrating that NO_3_^−^ and NH_4_^+^ exert distinct effects on cell wall synthesis in *M. importuna*.

### ﻿Consistency between transcriptome, proteome and metabolome

Analysis of concordance between gene expression at the transcriptional and translational levels identified 298 genes that were detected in both the transcriptomic (DEGs) and proteomic (DEPs) datasets. These genes demonstrated a Pearson’s correlation coefficient of −0.134 (*P* = 0.02) (Suppl. material 2), indicating minimal concordance between the transcriptome and proteome data. Transcriptome analysis highlighted pathways, such as amino sugar and nucleotide sugar metabolism, glutathione metabolism, peroxisome, fatty acid degradation and biosynthesis and ABC transporters. This diverged from the proteomic results, which highlighted N, tyrosine metabolism, thiamine metabolism, starch and sucrose metabolism, the phosphatidylinositol signalling pathway, N-glycan biosynthesis, ribosome biogenesis and RNA transport, while metabolomic analysis emphasised tyrosine metabolism, the biosynthesis of secondary metabolites, amino acids and biotin metabolism. This discrepancy may be attributed to temporal delays in translation, protein synthesis and post-translational modifications, as well as the metabolite compensation mechanism. Notably, both proteomic and metabolomic data revealed tyrosine metabolism to be the most enriched pathway, underscoring its pivotal role in regulating the response of *M. importuna* to different N forms. Accordingly, the functional modules identified by transcriptomic, proteomic and metabolomic data collectively demonstrated the effect of N on physiological activities in *M. importuna*, including C, fatty acid, amino acid metabolism, oxidative-stress response, cell fate determination and N metabolism.

### ﻿Physiological parameters of *M. importuna* are changed by N forms

Compared to the NH_4_^+^ treatment, NR, NiR and GOGAT activities were significantly increased in the NO_3_^−^ treatment (all *P* < 0.001) (Table 1). In contrast, GS and NADP-GDH activities were decreased by NO_3_^−^ (*P* < 0.001). We also assessed the activities of several key enzymes involved in carbohydrate degradation in *M. importuna*, such as cellulase, glucose dehydrogenase, xylanase, laccase, manganese peroxidase, lignin peroxidase and glucoamylase. Amongst them, the activities of cellulase, laccase, manganese peroxidase, lignin peroxidase and glucoamylase were higher (all *P* < 0.05) in NO_3_^−^ than in NH_4_^+^. This suggested that growth on NO_3_^−^ increased the lignocellulosic decomposition and starch hydrolysis capabilities of *M. importuna*, whereas xylanase activity appeared to be an exception, as it was reduced by NO_3_^−^ treatment (*P* = 0.024). Protease activity against NO_3_^−^ was lower than that against NH_4_^+^ (*P* = 0.002), indicating that NO_3_^−^ reduced the protein degradation ability of *M. importuna*. N forms had no effect on glucose dehydrogenase activity (*P* > 0.05). In addition, tyrosinase activity was increased by NO_3_^−^ (*P* < 0.001).

**Table 1.** Effects of NO_3_^−^-N (NN) and NH_4_^+^-N (AN) treatments on enzymatic activity and accumulation of metabolites related to cellular oxidative stress in *Morchella importuna* cultures.

	NN (n = 5)	AN (n = 5)	*P*-value
Enzymatic activity (nmol/min/g)			
Nitrate reductase	2167.4 ± 174.9	1432.1 ± 26.3	< 0.001
Nitrite reductase	1611.9 ± 184.1	276.3 ± 19.8	< 0.001
Glutamate synthase	6850.9 ± 3367.3	2760.8 ± 1556.7	0.039
Glutamine synthetase	5066.1 ± 695.5	6190.8 ± 877.3	0.020
NADP-Glutamate dehydrogenase	389.3 ± 48.5	679.9 ± 52.2	< 0.001
Cellulase	5717.4 ± 142.8	5487.9 ± 56.2	0.019
Xylanase	146.7 ± 9.6	164.6 ± 10.6	0.024
Laccase	313.2 ± 17.7	235.7 ± 3.1	< 0.001
Manganese peroxidase	163.4 ± 12.0	118.5 ± 7.8	< 0.001
Lignin peroxidase	63.4 ± 1.9	58.0 ± 2.9	0.011
Glucoamylase	17546.7 ± 290.3	16539.7 ± 404.2	0.002
Glucose dehydrogenase	702.3 ± 46.6	646.2 ± 25.8	0.055
Protease	42.4 ± 1.2	48.2 ± 2.3	0.002
Tyrosinase	6635.3 ± 636.1	1488.6 ± 189.5	< 0.001
Oxidative stress-related substances (nmol/g)			
Superoxide anion	5.8 ± 0.2	3.6 ± 0.1	< 0.001
Hydrogen peroxide	2394.7 ± 35.3	1567.2 ± 47.3	< 0.001
Malondialdehyde	21.2 ± 1.1	12.4 ± 0.7	< 0.001

We examined the accumulation of harmful substances associated with cellular oxidative stress in *M. importuna* mycelia and found that the levels of malondialdehyde (*P* < 0.001) and reactive oxygen species (H_2_O_2_ and O_2_^−^) (all *P* < 0.001) were higher in NO_3_^−^ than in NH_4_^+^ (Table 1). These findings suggest that NO_3_^−^ induced oxidative stress occurs in *M. importuna*.

## ﻿Discussion

NO_3_^−^ and NH_4_^+^ are the two primary inorganic N sources for fungi (Chen et al. 2021; Liu et al. 2022; Gao et al. 2025). These two forms of N differentially regulate mycelial growth and fruiting body formation in soil-grown mushrooms such as morels, an ascomycete (Carrasco et al. 2018; Tan et al. 2021b; Li et al. 2024; Noble et al. 2024; Zhang et al. 2024a). Multi-omics analyses were used to elucidate the distinct response mechanisms of *M. importuna* mycelium to NO_3_^−^ or NH_4_^+^ as the sole N source. Divergent effects of NO_3_^−^ and NH_4_^+^ on the regulation of N assimilation pathways and mycelial homeostasis were identified. The GS/GOGAT cycle and NADP-GDH pathway are the two major N assimilation pathways for fungi (Martin et al. 1986; Martin et al. 1994), both of which lead to the formation of glutamate, which not only participates in amino acid metabolism, but also acts as a signalling molecule to regulate different pathways (Walker and van der Donk 2016). NADP-GDH has been identified as the primary enzyme involved in glutamate formation in ascomycetous fungi, including several yeasts (Burn et al. 1974), as well as in *Aspergillus nidulans* and *Neurospora crassa* (Pateman 1969).

In the mushroom-forming fungus *Hebeloma cylindrosporum*, the expression of *NR* and *NiR* genes was repressed by NH_4_^+^ and strongly stimulated by NO_3_^−^ (Jargeat et al. 2003). As expected, transcriptomics, proteomics and enzyme activity tests revealed that NO_3_^−^ induced the expression and activity of NR and NiR in *M. importuna*, whereas it was suppressed by NH_4_^+^. Different N assimilation pathways were initiated by NH_4_^+^ and NO_3_^−^ in *M. importuna* mycelia. NO_3_^−^ inhibited both the gene expression and enzymatic activity of NADP-GDH while simultaneously increasing the levels of metabolite glutamate. Similarly, NH_4_^+^ increased the NADP-GDH activity in ascomycetes *N. crassa* and *A. nidulans* and decreased intracellular concentration of glutamate (Pateman 1969). This indicated that the GS/GOGAT cycle played a key role in glutamate synthesis in *M. importuna* when NO_3_^−^ was the main N source, as confirmed by the upregulation of GOGAT enzyme activity and the downregulation of GS enzyme activity (Martin et al. 1994). The change in glutamate levels could interfere with the tricarboxylic acid cycle and affect the energy regulation and cell homeostasis of *M. importuna* cells (Flores and Herrero 1994; Maechler et al. 2006; Wullschleger et al. 2006). Moreover, NO_3_^−^ as the sole N source, enhanced the lignocellulose decomposition and starch hydrolysis capabilities of *M. importuna*, while diminishing its protein degradation ability. These findings suggest that the distinct N metabolic pathways initiated by N in oxidised and reduced states triggers different cellular homeostasis, which subsequently influences the growth, development and morphogenesis of *M. importuna*.

In comparison to the direct assimilation of NH_4_^+^ in fungi, NO_3_^−^ uptake and assimilation involve additional transport and reduction steps, resulting in higher assimilation costs (Morton and Macmillan 1954; Patterson et al. 2013). Proteomics has found that the expression of numerous ribosomal proteins is repressed in *M. importuna* under NO_3_^−^ supplementation, likely impeding ribosome biogenesis and consequently inhibiting RNA transport and the expression of related proteins. This suggests that *M. importuna* experiences a state similar to N starvation stress when NO_3_^−^ serves as the sole N source. This explains the phenotypic phenomenon observed in this study, where the maximum biomass of *M. importuna* in NO_3_^−^ was lower than that in NH_4_^+^. Transcriptomic enrichment of glutathione metabolism and peroxisome pathways suggested that NO_3_^−^ induced oxidative stress in *M. importuna*. Correspondingly, metabolomics confirmed that the increased glutamate metabolism pathway under NO_3_^−^ treatment induced the downstream glutathione metabolism pathway, thereby elevating the levels of reduced glutathione and its intermediate, gamma-glutamylcysteine, which functioned as a major antioxidant to prevent cell damage caused by various oxidative stressors (Quintana-Cabrera et al. 2012). Furthermore, NO_3_^−^ was observed to induce the accumulation of malondialdehyde and ROS. These findings indicate that *M. importuna* mycelia respond to NO_3_^−^-induced N starvation-like phenomenon by initiating oxidative stress. N depletion has been shown to cause the production of ROS in microbial cells and ROS-induced DNA-damage repair is one of the primary driving forces of sexual reproduction in all organisms (Hörandl 2009; Kurihara et al. 2012; Wallen and Perlin 2018). Similar phenomena are frequently observed in traditional edible mushroom cultivation techniques, such as inducing oxidative stress through mechanical wounds caused by mycelial scratching, which stimulates the transition from mycelial growth to reproductive growth (Xu et al. 2023). Consequently, NO_3_^−^-induced oxidative stress can be one of the key factors that promote sexual morphogenesis and fruiting body formation in *M. importuna*.

Previous studies have suggested that melanin, which relies on tyrosinase activity, is associated with the formation of fungal fruiting bodies (Langfelder et al. 2003; Teichert and Nowrousian 2011; Pöggeler et al. 2018). Melanin biosynthesis in *N. crassa* (Prade et al. 1984) and *Tuber* species (Ragnelli et al. 1992) is correlated with the reproductive cycle. Moreover, melanin gene expression is significantly repressed in submerged cultures, in which reproductive development does not occur. For instance, sterile mutants of *Sordaria macrospora* exhibited reduced transcript levels of melanin biosynthesis genes (Engh et al. 2007). Similarly, mutants of *Ophiostoma piliferum* and *Podospora anserina* with defects in melanin biosynthesis displayed impaired fruiting body formation (Gautier et al. 2021). In the present study, both proteomics and metabolomics found that the enrichment of the tyrosine metabolic pathway was particularly pronounced after NO_3_^−^ treatment, and the corresponding melanin synthesis substrates, such as L-tyrosine and L-dopa, as well as several tyrosinase proteins and overall tyrosinase activity, were upregulated. This indicated that NO_3_^−^ treatment accelerated melanin deposition in *M. importuna*, which stabilised the cell wall, thereby protecting the cells from extreme environmental conditions and pathogen infections, facilitating the reproductive development of *Morchella* (Cordero and Casadevall 2017).

Genes involved in cell wall biogenesis and metabolism also contribute to the coordinated development of fruiting bodies in fungi (Pöggeler et al. 2018; Virágh et al. 2022). In this study, transcriptomics and proteomics revealed that NO_3_^−^ treatment activated the pathways regulating cell wall synthesis in fungi, including amino sugar and nucleotide sugar metabolism and N-glycan synthesis. Previous research has demonstrated that the N-glycans are essential for the integrity of the cell wall of *Saccharomyces cerevisiae* (Orlean 2012). Furthermore, the N-glycosylation is involved in regulating the growth and morphogenesis of fungi (Chen et al. 2020; Mei et al. 2023). In the current study, the upregulation of fructose and glucokinase genes induced the abundance of the precursor substance D-glucosamine 6-phosphate (GlcN-6-P) of N-acetyl-D-glucosamine (GlcNAc). As a core component of N-glycans, GlcNAc is a key constituent of fungal cell wall chitin (Min et al. 2019). Additionally, the nucleotide sugar UDP-GlcA, a precursor substance of cell wall polymers, such as galacturonic acid and xylose (Kanter et al. 2005; Reboul et al. 2011), was also upregulated in NO_3_^−^. This observation indicated that NO_3_^−^ activated the glycosylation level of *M. importuna* and these complex matrices composed of polysaccharides and glycoproteins further form the cell wall, creating a protective barrier and promoting cell adhesion (Traeger and Nowrousian 2015), which is crucial for the morphogenesis of *M. importuna*.

This investigation demonstrated low concordance amongst the transcriptome, proteome and metabolome data. One potential explanation for this phenomenon is that different N forms influence the ribosomal binding sites, resulting in inconsistent mRNA translation efficiencies (Chen et al. 2024). Proteomic analysis revealed specific enrichment of the ribosome biogenesis pathway under NO_3_^−^ treatment, lending support to this hypothesis. An alternative explanation may be the post-translational modifications of proteins in *M. importuna* induced by different N forms (Chen et al. 2023). We observed specific enrichment of the N-glycan pathway in NO_3_^−^ at the protein level, which could lead to glycosylation modifications of proteins post-translation, thereby altering protein abundance and physiological functions. Additional research is necessary to elucidate the glycosylation modifications of *Morchella* proteins induced by different forms of N and their role in the regulation of physiological functions.

## ﻿Conclusions

Our results indicate that NO_3_^−^ and NH_4_^+^ initiate different N assimilation pathways in morels. Compared with NH_4_^+^, supplying NO_3_^−^ as the sole N source increased glutamate levels in *M. importuna* through the GS/GOGAT cycle rather than the NADP-GDH pathway. NO_3_^−^ induced a status analogous to N starvation and increased ROS levels in *M. importuna* mycelia, subsequently activating pathways, such as glutathione metabolism, tyrosine metabolism and N-glycan synthesis to mitigate adverse stress conditions. These activated pathways are associated with increased antioxidant levels, melanin deposition and cell wall formation and are also implicated in the regulation of sexual morphogenesis and fruiting body formation in macrofungi. A limitation of this study is the absence of direct evidence for the regulation of sexual morphogenesis in *M. importuna* by N forms. Future studies should integrate the stages of mycelial growth, primordium formation and fruiting body development to further elucidate the regulatory mechanisms of nitrogen forms in *M. importuna* fruiting morphogenesis. The findings of this study demonstrate NO_3_^−^ and NH_4_^+^ shape different homeostasis and physiological states of *Morchella* by inducing different responses to N mechanisms. This provides insights into the molecular mechanisms by which N forms regulate the growth and development of macrofungi and offers theoretical support for increasing macrofungi productivity through optimised application of different N forms.

## ﻿Additional information

### Use of AI

No use of AI was reported.

### Adherence to national and international regulations

All the fungal strains used in this study have been legally obtained, respecting the Convention on Biological Diversity (Rio Convention).

### Author ORCIDs

Yang Yu https://orcid.org/0000-0002-1443-8543

Francis M. Martin https://orcid.org/0000-0002-4737-3715

Hao Tan https://orcid.org/0000-0002-4382-5470

### Data availability

The transcriptomic data in this article have been deposited in the NCBI databank under the accession number PRJNA1211872. The mass spectrometry proteomics data were deposited in the ProteomeXchange Consortium with the dataset identifier PXD059958.

## Supplementary materials

Supplementary material 1 Pearson correlation between transcriptome and proteome data in this study Data type pdf https://binary.pensoft.net/file/1506245This dataset is made available under the Open Database License (http://opendatacommons.org/licenses/odbl/1.0/). The Open Database License (ODbL) is a license agreement intended to allow users to freely share, modify, and use this Dataset while maintaining this same freedom for others, provided that the original source and author(s) are credited.Yang Yu, Tianhai Liu, Jing Li, Xiang Wu, Shengyin Zhang, Yong Wang, Jie Tang, Weihong Peng, Francis M. Martin, Hao Tan

Supplementary material 2 Supplementary image Data type pdf https://binary.pensoft.net/file/1506246This dataset is made available under the Open Database License (http://opendatacommons.org/licenses/odbl/1.0/). The Open Database License (ODbL) is a license agreement intended to allow users to freely share, modify, and use this Dataset while maintaining this same freedom for others, provided that the original source and author(s) are credited.Yang Yu, Tianhai Liu, Jing Li, Xiang Wu, Shengyin Zhang, Yong Wang, Jie Tang, Weihong Peng, Francis M. Martin, Hao Tan

Supplementary material 3 Bioinformatics Data type xlsx https://binary.pensoft.net/file/1506247This dataset is made available under the Open Database License (http://opendatacommons.org/licenses/odbl/1.0/). The Open Database License (ODbL) is a license agreement intended to allow users to freely share, modify, and use this Dataset while maintaining this same freedom for others, provided that the original source and author(s) are credited.Yang Yu, Tianhai Liu, Jing Li, Xiang Wu, Shengyin Zhang, Yong Wang, Jie Tang, Weihong Peng, Francis M. Martin, Hao Tan
