# Supplementary figures and images for: ﻿Multi-omics reveals nitrate-induced oxidative stress and morphogenesis pathways in Morchella importuna
Source: IMA Fungus. 2026 Jan 6;17:e159999. doi: 10.3897/imafungus.17.159999 (PMC12800781; doi:10.3897/imafungus.17.159999)

$\text{NO}_3^-$ -N

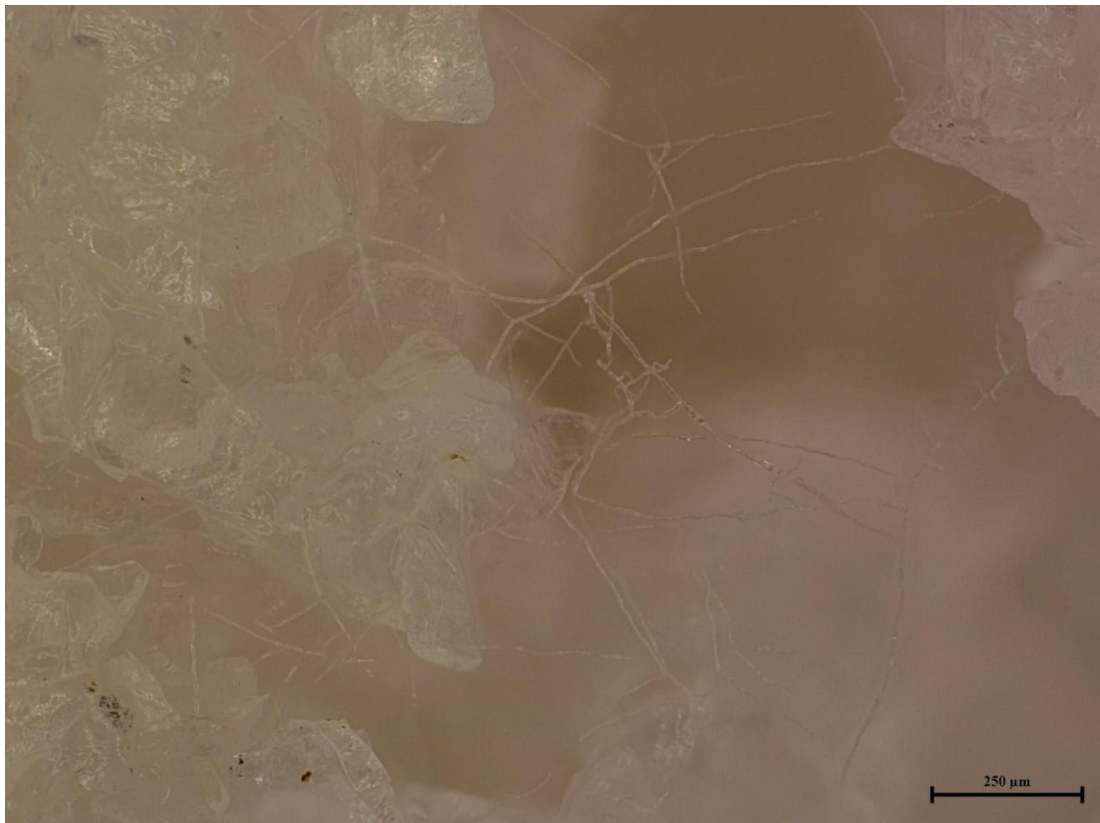

$\text{NH}_4^+$ -N

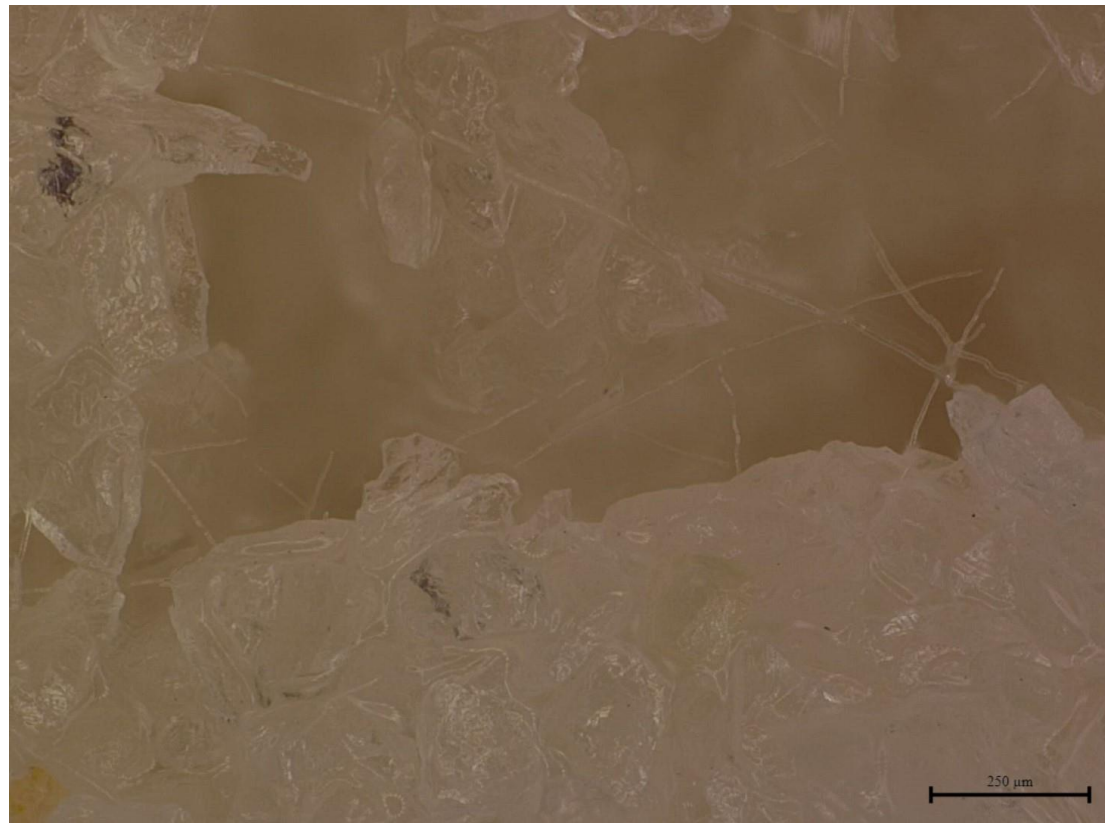

Supplement: Supplementary material 1 — Pearson correlation between transcriptome and proteome data in this study [file imafungus-17-e159999-s001.pdf]

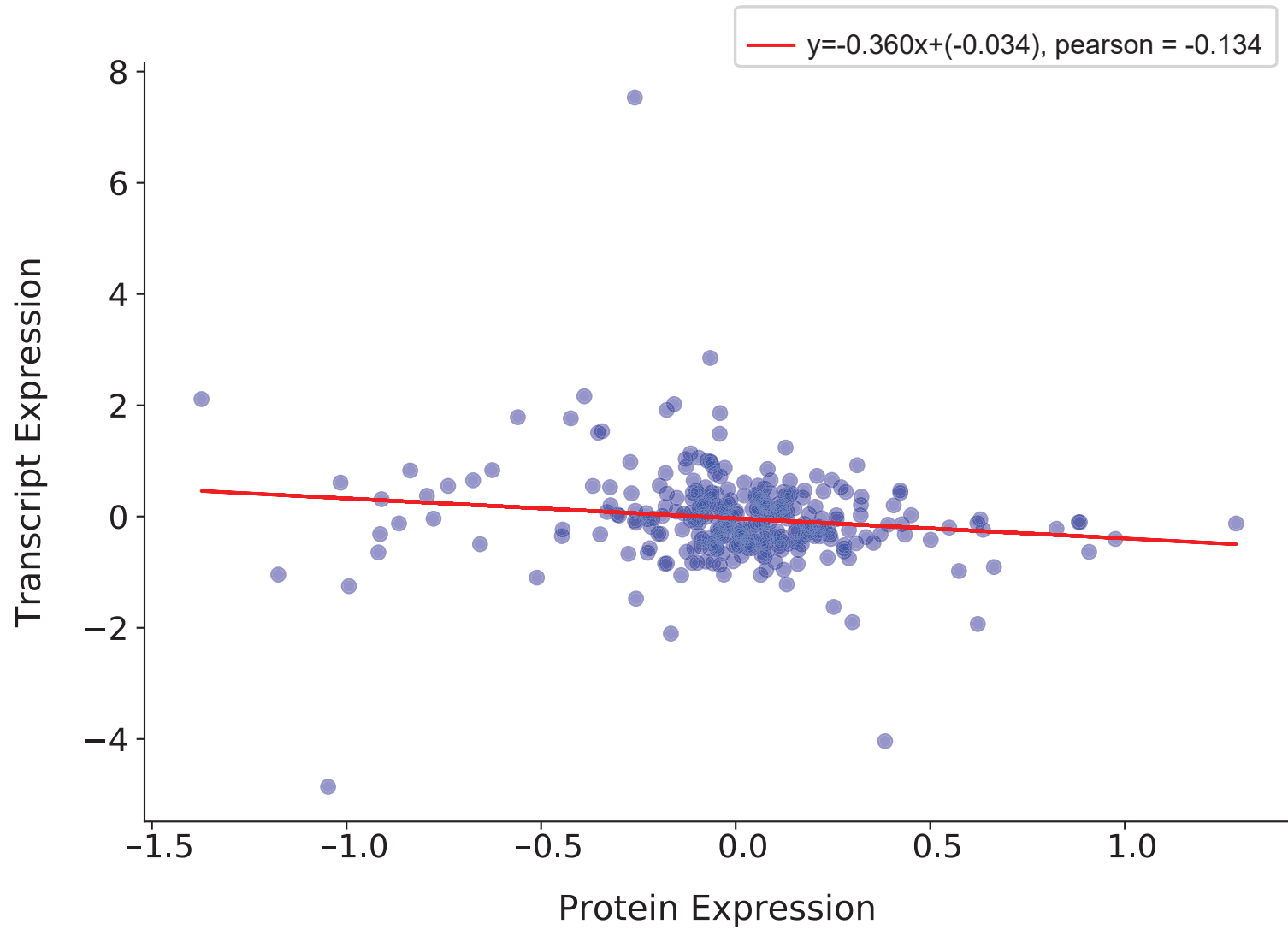

Supplement: Supplementary material 2 — Supplementary image [file imafungus-17-e159999-s002.pdf]
